# Molten Salt Synthesis and Electrochemical Evaluation of Na/Ag-Containing Mn_x_O_y_ Composites for Pseudocapacitor Applications

**DOI:** 10.3390/ma18163869

**Published:** 2025-08-18

**Authors:** Carmen Martínez-Morales, Antonio Romero-Serrano, Josué López-Rodríguez, Paulina Arellanes-Lozada

**Affiliations:** 1 Departamento de Metalurgia y Materiales, Instituto Politécnico Nacional-ESIQIE, UPALM-Zacatenco, Av. Instituto Politécnico Nacional s/n, Lindavista, G. A. Madero, CDMX, Ciudad de Mexico 07738, Mexico; kar.mtz.m@gmail.com (C.M.-M.); aromeros@ipn.mx (A.R.-S.); 2 Dirección de Investigación, Instituto Mexicano del Petróleo. Eje Central Norte Lázaro Cárdenas No. 152, San Bartolo Atepehuacan, G. A. Madero, CDMX, Ciudad de Mexico 07730, Mexico

**Keywords:** molten salt synthesis, EIS, sodium birnessite, silver hollandite

## Abstract

Different composites of manganese oxides (Mn_*x*_O_*y*_) containing sodium (Na) and silver (Ag) were synthesized by the molten salt method with various MnSO_4_·H_2_O/NaNO_3_ (M/N) molar ratios (between 0.3 and 1), and different AgNO_3_ and NaOH amounts, obtaining two groups of materials: without the addition of AgNO_3_ (labeled as M/N) and with AgNO_3_ (labeled as M/N-A). As for the M/N group, the system with the lowest M/N ratio yielded the highest specific capacitance (160.5 F $g^{-1}$), attributed to the formation of Mn_3_O_4_ and sodium birnessite. In the M/N-A group, the 1 M/N-0.5A system, produced with M/N ratio of 1 and addition of 0.5 g of AgNO_3_, exhibited the highest specific capacitance (229.1 F $g^{-1}$), associated with the presence of Mn_2_O_3_, silver hollandite, and metallic Ag. This enhancement is attributed to the synergistic effects of Na^+^ and Ag^+^ ions, which improve charge transfer kinetics and electrochemical performance. It was demonstrated that decreasing the MnSO_4_·H_2_O/NaNO_3_ ratio in the M/N group and increasing AgNO_3_ content in the M/N-A group enhances the electrochemically active surface area. Galvanostatic charge–discharge (GCD) and electrochemical impedance spectroscopy (EIS) techniques confirmed that the 1 M/N-0.5A system exhibited the best performance, characterized by high energy retention, stable cycling behavior, and low capacitance dispersion, indicating its strong potential as an active material for pseudocapacitor applications.

## 1. Introduction

Currently, the world is facing a significant energy crisis triggered by the accelerated demographic growth and over-exploitation of non-renewable energy sources such as fossil fuels [1,2]. Before this challenge, the scientific community had focused its efforts on developing and optimizing advanced energy storing technologies that include electrochemical batteries, fuel cells, and supercapacitors in order to improve the efficiency, sustainability, and feasibility of the energy systems [3,4]. Supercapacitors (SCs) are storing systems of electrochemical energy that follow a Faradaic process based on redox reactions on the electrode material, and due to their nature, these materials have high power, energy density, and good cyclability capacity [5,6]. There are two types of SCs: (a) the electric double-layer capacitors, which accumulate and separate charge at the electrode–electrolyte interface without involving electrochemical reactions and (b) pseudocapacitors, which store charge through redox reactions or insert ions between the electrode and electrolyte by means of their high power density, fast charge–discharge response, and good electrochemical stability [7,8]. The different types of pseudocapacitors can be identified by their chemical nature, which can be represented by transition metal oxides (${\mathrm{MO}}_{x}$; M = Fe, Co, Ni, Mn, etc.), metal hydroxides, and conducting polymers [9,10]. Numerous transition metal oxides have been employed as pseudocapacitors, where ${\mathrm{RuO}}_{x}$, ${\mathrm{MnO}}_{x}$, NiO, ${\mathrm{CoO}}_{x}$, ${\mathrm{MoO}}_{x}$, and ${\mathrm{VO}}_{x}$ have stood out [11,12]. ${\mathrm{MnO}}_{x}$ materials such as MnO, ${\mathrm{MnO}}_{2}$, ${\mathrm{Mn}}_{2}$$O_{3}$, ${\mathrm{Mn}}_{3}$$O_{4}$, etc., have been widely studied because of their high theoretical capacitance (755–1232 mAh $g^{-1}$) [13]. ${\mathrm{MnO}}_{2}$ and ${\mathrm{Mn}}_{2}$$O_{3}$ stand out due to characteristics, such as high theoretical specific capacitance, excellent reversibility, high specific surface area, relatively low conversion potential, small voltage hysteresis, multi-oxidation states, low cost, high availability, and ecofriendly nature [2,5,7,9,13,14]. However, these ${\mathrm{Mn}}_{x}$$O_{y}$ materials have a low electrical conductivity compared to others electrochemical capacitors [5,13,14]. One strategy to overcome their kinetic and conductivity limitations is to insert alkyl cations such as Na, K, Ag, and transition metals into the ${\mathrm{Mn}}_{x}$$O_{y}$ crystal lattice, thereby improving the material’s electrochemical behavior [12]. Thanigai et al. performed a comparative analysis of the electrochemical behavior of ${\mathrm{Mn}}_{3}$$O_{4}$, ${\mathrm{Na-MnO}}_{2}$, and ${\mathrm{Na-MnO}}_{2-x}$; the latter was obtained by introducing oxygen vacancies in ${\mathrm{Na}}^{+}$ ions inserted in ${\mathrm{MnO}}_{2}$. They concluded that the improvement of electrochemical properties of materials is related to electrochemical kinetics and the increase in the active surface area of ${\mathrm{Na-MnO}}_{2-x}$ [9]. Vashisth et al. conducted a study using the solid-state reaction method to synthesize α${\mathrm{-Na}}_{0.5}$Mn_0.9302_@Na_0.91_MnO_2_ (NaMnO) and $K_{0.48}$Mn_1.9405_@Na_0.91_MnO_2_ (KNaMnO) nanocomposites; they indicated that no Na-K phases were formed; rather, these cations interacted independently with the ${\mathrm{MnO}}_{2}$ crystal lattice, which generated high specific capacitance values for NaMnO and KNaMnO of approximately 361 and 143 F $g^{-1}$, respectively [15]. In contrast, the synthesis of ${\mathrm{Ag}}_{2}{\mathrm{-MnO}}_{2}$ ultrathin nanosheets has also been studied by Rahman et al., in 2021, who indicated that one-pot hydrothermal synthesis process at 150 °C can favor the electrode–electrolyte diffusive process by the good electrical conductivity of Ag ions, and the increased active sites at the boundaries of the ${\mathrm{MnO}}_{2}$ crystal lattice [12].

It is well known that the properties and performance of materials are greatly affected by the synthesis method and conditions used to produce them. Among the different synthesis methods for obtaining electrode materials such as hydrothermal [16,17,18], sol-gel [19], co-precipitation [20], microemulsion [21], and combustion, the molten salt synthesis has proven to be a cost-effective, simple to operate, easy to scale, generalizable, and environmentally friendly process [16,22,23]. Additionally, molten salt synthesis has certain advantages over other methods, such as solid-state reactions. With a liquid phase of salts, faster mass transport by convection and diffusion is achieved [23,24,25]. This is evident in the study conducted by Zhao et al., who synthesized ${\mathrm{Mn}}_{2}O_{3}$ by the molten salt method employing ${\mathrm{KNO}}_{3}{\mathrm{-NaNO}}_{2}{\mathrm{-NaNO}}_{3}$ and manganese acetate as reaction medium and manganese source, respectively; these authors claimed that such a synthesis method could achieve electrode materials with outstanding electrochemical performance related to a high specific surface area [22].

In this work, ten electrode materials were synthesized by the molten salt method, modifying the MnSO_4_·$H_{2}{\mathrm{O/NaNO}}_{3}$ ratio and ${\mathrm{AgNO}}_{3}$ concentration, in order to obtain ${\mathrm{Mn}}_{x}O_{y}$ materials with ${\mathrm{Na}}^{+}$ and ${\mathrm{Ag}}^{+}$ residual ions that improve the pseudocapacitive properties of ${\mathrm{Mn}}_{x}O_{y}$. X-ray diffraction (XRD) and scanning electron microscopy–energy dispersive X-ray spectroscopy (SEM-EDS) were used to analyze the morphology and crystalline structure of the electrode materials, whereas the performance of the electrodes was evaluated using the cyclic voltammetry (CV), galvanostatic charge–discharge (GCD), and electrochemical impedance spectroscopy (EIS) techniques with 0.1 M ${\mathrm{Na}}_{2}{\mathrm{SO}}_{4}$ as electrolyte.

## 2. Materials and Methods

### 2.1. Synthesis

Ten electrode materials were synthesized by the molten salt method, whose abbreviations and compositions are presented in Table 1. All the reagents used in the experiments were of analytical grade and used without further purification. Different mass ratios of MnSO_4_·H_2_O, ${\mathrm{NaNO}}_{3}$, ${\mathrm{AgNO}}_{3}$, and NaOH were placed in an agate mortar, and ground and homogenized for 30 min to promote interaction between the reactants during precipitation. Subsequently, a nickel crucible containing each salt mixture (MnSO_4_·H_2_O/NaNO_3_AgNO_3_/NaOH) was put in a tube furnace supplied with Ar at 420 °C for 30 min, at a heating rate of 10 °C/min. Once the mixed salt reached the melting point, the samples were cooled down inside the furnace until they reached room temperature. Then, the precipitated powder was collected, washed, and centrifuged with deionised water eight times [26,27]. This procedure was carried out to remove sub-products of the molten salt synthesis (i.e., remaining water-soluble salts) and separate the ${\mathrm{Mn}}_{x}O_{y}$ composites. After drying them in an oven at 90 °C for 6 h, a black solid was finally obtained.

### 2.2. Characterization of Materials

XRD analysis of the different materials was carried out using a Bruker D8 Advance diffractometer within a 2θ interval ranging from 0 to 90° and Cu kα radiation with a wavelength of approximately λ = 1.54056 Å. The XRD results were processed using the Match software version 4.1 Build 311. The surface morphology and elemental composition of the materials were analyzed by SEM using a high-resolution microscope JEOL JSM 6701F coupled with EDS. ImageJ software Version 1.54f (Wayne Rasband, MD, USA) was used to analyze the particle size distribution on the surface of the ${\mathrm{Mn}}_{x}O_{y}$ composites. The electrochemical behavior of the synthesized materials was analyzed by CV, GCD, and EIS, employing a Biologic SP-300 potentiostat/galvanostat (BioLogic Science Instruments, Seyssinet-Pariset, France) and the EC-Lab software version V11.34. Electrochemical measurements were performed in a three-electrode glass cell with Ag/AgCl/NaCl [3 M] as reference electrode, graphite as counter electrode, and ${\mathrm{Mn}}_{x}O_{y}$/glassy carbon as working electrode. The latter was prepared by using ${\mathrm{Mn}}_{x}O_{y}$-based ink on a glassy carbon substrate. The ink was made by mixing 0.03 g of synthesized composites, 0.0015 g of carbon black vulcan XC 72, and 300 µL of acetone with 60 µL of a 5 wt.% Nafion 117 solution. The mixture was placed in an ultrasonic bath for 30 min to form a homogeneous black suspension, which was then carefully pipetted onto a polished glassy carbon electrode of 3 mm in diameter using 1000 and 2000 grit SiC paper and diamond paste until achieving a mirror finish to ensure proper adhesion of the materials. The mass loading of Na/Ag-containing ${\mathrm{Mn}}_{x}O_{y}$ electrodes was 0.279 mg. Finally, the ink was dried at room temperature for 1 h [28,29]. The employed electrolyte was a 0.1 M ${\mathrm{Na}}_{2}{\mathrm{SO}}_{4}$ solution. CV measurements were conducted within a potential interval ranging from 0.0 to 0.9 V vs. Ag/AgCl/NaCl [3 M] at scan rates of 5, 10, 20, 50, and 100 mV $s^{-1}$. GCD profiles were obtained at current densities of 0.5, 1.0, 1.5, and 2.0 A $g^{-1}$ within a potential range of 0.0 to 1.0 V. Cycling performance was studied at a scan rate of 1 A $g^{-1}$ for over 500 cycles. Regarding the EIS technique, experimental tests were run at open-circuit potential within a frequency interval ranging from 100 kHz to 10 mHz with sine wave amplitude of 10 mV.

## 3. Results

### 3.1. Materials Characterization Using XRD and SEM-EDS

**Figure 1:**
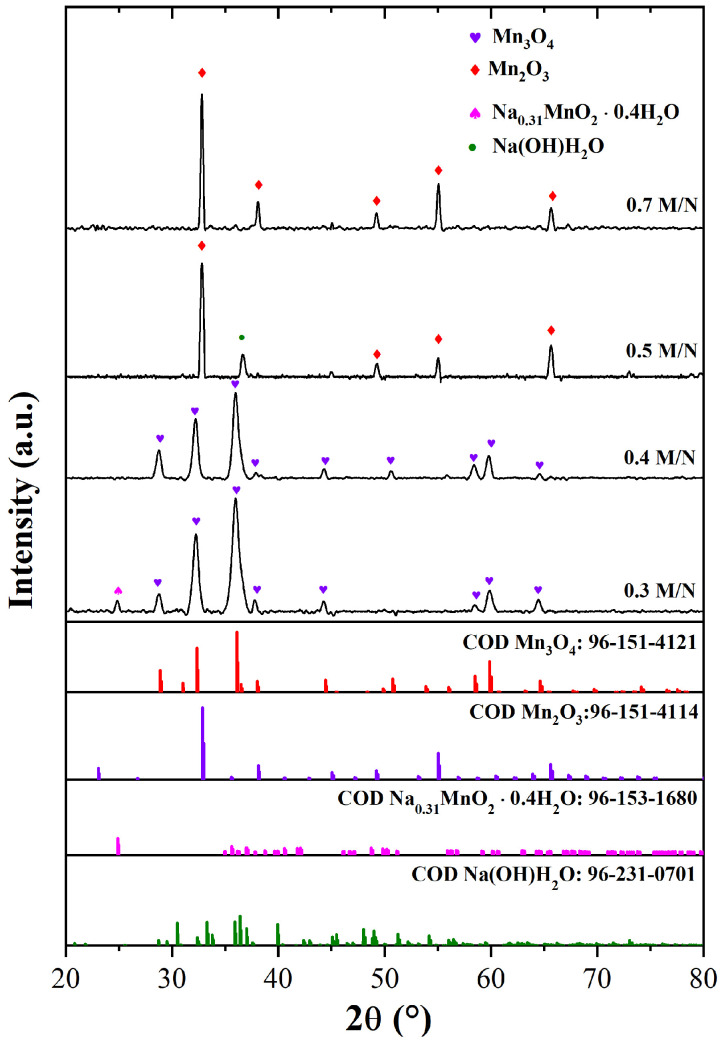
XRD pattern of Na-containing ${\mathrm{Mn}}_{x}O_{y}$ composites: 0.3 M/N, 0.4 M/N, 0.5 M/N, and 0.7 M/N.

Figure 1 shows the XRD spectra of Na/Ag-containing ${\mathrm{Mn}}_{x}O_{y}$ composites synthesized by the molten salt method with different MnSO_4_·H_2_O/NaNO_3_ ratios (M/N). The 0.5 M/N and 0.7 M/N spectra show characteristic peaks at 2θ of 23.1, 32.8, 38.1, 55.0, and 65.$6^{\circ }$, indicating the formation of ${\mathrm{Mn}}_{2}O_{3}$ (Bixbyite) (COD 96-151-4114) [30]; this compound has a cubic phase and lattice parameter of a = 9.43000 Å [30,31]. This crystal phase, obtained by hydrothermal synthesis, has been reported to exhibit good electrocatalytic behavior [31]. Regarding the 0.5 M/N system, an additional peak at 36.$7^{\circ }$ was associated with the formation of Na(OH)($H_{2}$O) (COD 96-231-0701), which could reduce the electrochemical behavior of the electrode material. In contrast, a new phase was formed when the M/N ratio decreased; this event occurred in the 0.3 M/N and 0.4 M/N systems, where ${\mathrm{Mn}}_{3}O_{4}$ (Hausmannita) was present as confirmed by the peaks at 28.8, 31.0, 32.2, 38.0, 44.4, 50.8, 58.5, 59.8, and 64.$6^{\circ }$ (COD 96-151-4121), see Figure 1 [32]. Firstly, the molten salt synthesis process allowed the ionization of ${\mathrm{MnSO}}_{4}$ (Reaction (1)). Additionally, the synthesis of ${\mathrm{Mn}}_{3}O_{4}$ was achieved thanks to the active oxygen released from the nitrate decomposition reaction (Reactions (2) and (3)) and the presence of hydroxyl ions from NaOH ionization, as observed in Reactions (4)–(6) [9,33]:

(1) $$\begin{array}{c} {\mathrm{MnSO}}_{4}\to {\mathrm{Mn}}^{2+}+{\mathrm{SO}}_{4}^{2+} \end{array}$$

(2) $$\begin{array}{c} 2{\mathrm{NaNO}}_{3}\to 2{\mathrm{NaNO}}_{2}+O_{2}↑ \end{array}$$

(3) $$\begin{array}{c} 2{\mathrm{AgNO}}_{3}\to 2\mathrm{Ag}+2{\mathrm{NO}}_{2}+O_{2}↑ \end{array}$$

(4) $$\begin{array}{c} 2{\mathrm{Mn}}^{2+}+5{\mathrm{OH}}^{-}\to \mathrm{Mn}{\left(\mathrm{OH}\right)}_{2}+2\mathrm{Mn}{\left(\mathrm{OH}\right)}_{3} \end{array}$$

(5) $$\begin{array}{c} \mathrm{Mn}{\left(\mathrm{OH}\right)}_{2}+2\mathrm{Mn}{\left(\mathrm{OH}\right)}_{3}\to {\mathrm{Mn}}_{3}O_{4}+4H_{2}O \end{array}$$

(6) $$\begin{array}{c} 6{\mathrm{Mn}}^{2+}+12{\mathrm{OH}}^{-}+O_{2}\to 2{\mathrm{Mn}}_{3}O_{4}+6H_{2}O \end{array}$$

${\mathrm{Mn}}_{3}O_{4}$ has a tetragonal arrangement and belongs to the spinel class, where ${\mathrm{Mn}}^{2+}$ and ${\mathrm{Mn}}^{3+}$ cations occupy tetrahedral and octahedral sites, respectively [32,34]. Therefore, manganese is surrounded by oxygen atoms at tetrahedral and octahedral positions [33]. Additionally, the 0.3 M/N system shows a characteristic peak at 24.$9^{\circ }$, related to ${\mathrm{Na}}_{0.31}^{1+}‐$(${\mathrm{Mn}}_{0.69}^{4+}{\mathrm{Mn}}_{0.31}^{3+}$)O_2_ · $0.4H_{2}$O (sodium birnessite) formation (COD 96-153-1680). This compound has also been obtained by hydrothermal synthesis and has a tricyclic structure when sodium cations and water molecules are distributed by vacancy-free Mn-bearing octahedral layers [35]. The presence of sodium birnessite could result in better electrochemical behavior due to the increased conductivity and/or active surface area with respect to the other systems [9,35].

**Figure 2:**
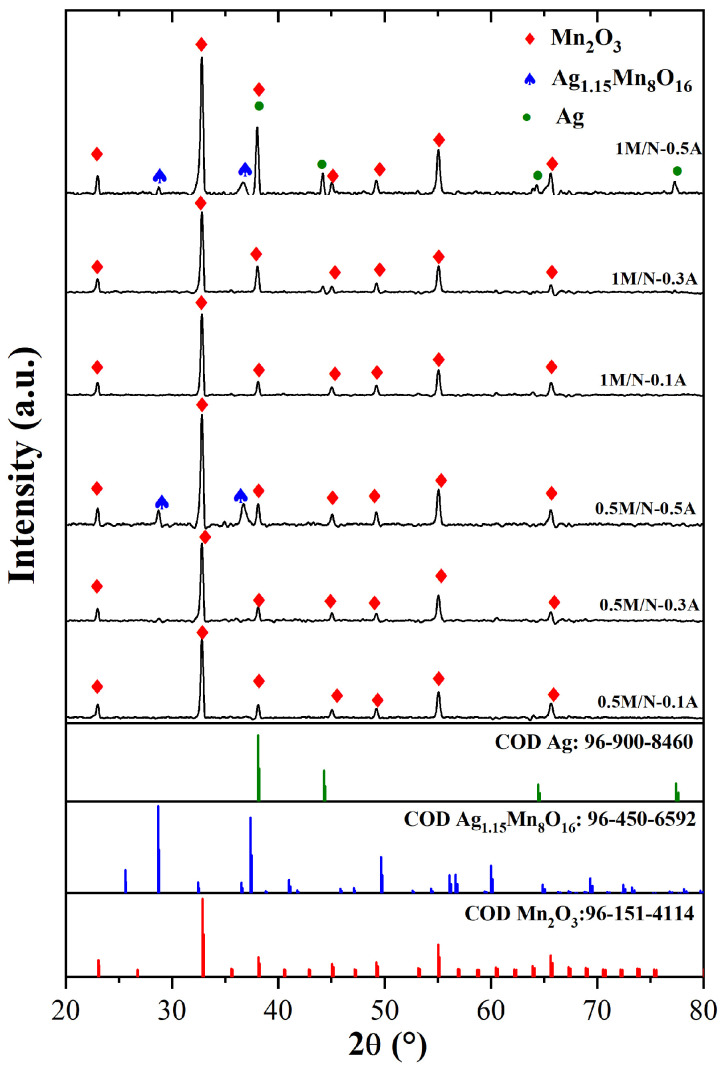
XRD pattern of Na/Ag-containing ${\mathrm{Mn}}_{x}O_{y}$ composites: 0.5 M/N-0.1A, 0.5 M/N-0.3A, 0.5 M/N-0.5A, 1 M/N-0.1A, 1 M/N-0.3A, and 1 M/N-0.5A.

Figure 2 shows XRD spectra of MnSO_4_·$H_{2}{\mathrm{O/NaNO}}_{3}$ systems synthesized with different concentrations of ${\mathrm{AgNO}}_{3}$ (M/N-A). All the samples presented peaks of the ${\mathrm{Mn}}_{2}O_{3}$ phase at 2θ of 23.1, 32.8, 38.1, 45.1, 49.2, 55.0, and 65.6 ° (COD 96-151-4114) [30]. The low concentration of ${\mathrm{AgNO}}_{3}$ in the 0.5 M/N-0.1A, 0.5 M/N-0.3A, 1 M/N-0.1A, and 1 M/N-0.3A systems did not promote the doping of Ag into the material lattice. Since the amount of NaOH was reduced to 1 g in electrode materials synthesized with ${\mathrm{AgNO}}_{3}$, the ${\mathrm{Mn}}_{2}O_{3}$ synthesis was favored by the reaction with oxygen released from the precursors (Reactions (2) and (3)) and $\mathrm{Mn}{\left(\mathrm{OH}\right)}_{2}$ (Reaction (7)) [36,37].

(7) $$\begin{array}{c} 4\mathrm{Mn}{\left(\mathrm{OH}\right)}_{2}+O_{2}\to 2{\mathrm{Mn}}_{2}O_{3}+4H_{2}O \end{array}$$

Additionally, XRD spectra of the 0.5 M/N-0.5A and 1 M/N-0.5A systems exhibited characteristic peaks of ${\mathrm{Ag}}_{1.15}{\mathrm{Mn}}_{8}O_{16}$ (silver hollandite) at 2θ of 28.7, 36.7, and 37.$4^{\circ }$ (COD 96-450-6592) [38]. Hollandite-type materials consist of ${\mathrm{MnO}}_{6}$ octahedra with a tunnel structure of approximately ∼4.7 Å, which facilitates the insertion of small ions such as ${\mathrm{Na}}^{+}$, $K^{+}$, ${\mathrm{Rb}}^{+}$, ${\mathrm{Ag}}^{+}$, and ${\mathrm{Ba}}^{2+}$ [39,40]. Particularly, silver hollandite is an electrochemically active material because ${\mathrm{Ag}}^{+}$ ions are located at the center of the unit cell, as reported by Takeuchi et al. [38]. Its synthesis by solid-state and hydrothermal methods is difficult to achieve, as reported by different research works [41,42,43,44]. However, in this study, better ionic diffusion of molten salts facilitated its production. The insertion of Ag atoms into the crystal lattice could enhance the electron transport efficiency and specific capacitance of the 0.5 M/N-0.5A and 1 M/N-0.5A systems due to the excellent electrical conductivity and surface activity of Ag atoms [12]. Finally, the XRD spectrum of the 1 M/N-0.5A system shows additional peaks at 2θ of 38.0, 44.2, 64.4, and 77.$4^{\circ }$, which are associated with Ag (COD 96-900-8460) [38]. Since the relative intensity of the Ag peaks is higher than that of the ${\mathrm{Ag}}_{1.15}{\mathrm{Mn}}_{8}O_{16}$ peaks, it can be suggested that the doping of ${\mathrm{Mn}}_{2}O_{3}$ with Ag atoms was favored due to the high and low concentration of ${\mathrm{AgNO}}_{3}$ and ${\mathrm{NaNO}}_{3}$ precursors, respectively.

**Figure 3:**
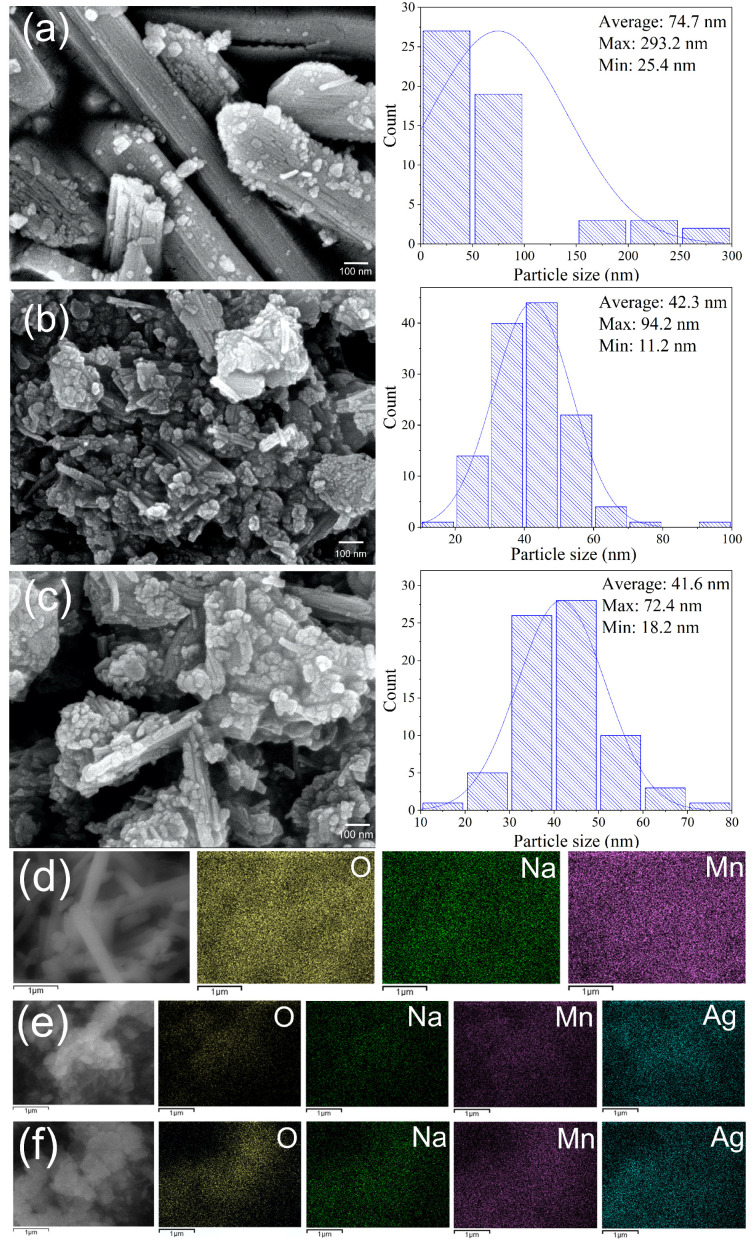
SEM micrographs at 80,000x and particle size histograms of Na/Ag-containing ${\mathrm{Mn}}_{x}O_{y}$ composites: (**a**) 0.3 M/N, (**b**) 0.5 M/N-0.5A, and (**c**) 1 M/N-0.5A. Elemental mapping of (**d**) 0.3 M/N, (**e**) 0.5 M/N-0.5A, and (**f**) 1 M/N-0.5A.

The surface morphology of the synthesized materials was analyzed by SEM-EDS (Figure 3 and Table 2). Figure 3a shows that the 0.3 M/N sample consists of disordered stacked bars of ${\mathrm{Mn}}_{3}O_{4}$ and sodium birnessite [45]. These bars reach a diameter of up to 293 nm and are covered with smaller particles, as shown in the corresponding histogram. The elemental mapping and EDS results indicate a uniform distribution of Mn, O, and Na, suggesting that the ${\mathrm{Na}}_{0.31}^{1+}$(${\mathrm{Mn}}_{0.69}^{4+}{\mathrm{Mn}}_{0.31}^{3+}$)O_2_ · $0.4H_{2}$O phase is dispersed uniformly. The 0.5 M/N-0.5A and 1 M/N-0.5A samples, synthesized with ${\mathrm{Ag/NO}}_{3}$, are presented in Figure 3b and c, respectively. Their surface morphology has particles of ${\mathrm{Mn}}_{2}O_{3}$, silver hollandite, and Ag of approximately 42 nm in size (see corresponding histogram) [46,47]. The elemental mapping reveals the uniform distribution of Ag, confirming the presence of ${\mathrm{Ag}}_{1.15}{\mathrm{Mn}}_{8}O_{16}$ and Ag. These results suggest that the molten salt synthesizing process enabled the nitrite and hydroxide salts to completely interact with the Mn source, thereby preventing product agglomeration.

### 3.2. Cyclic Voltammetry

**Figure 4:**
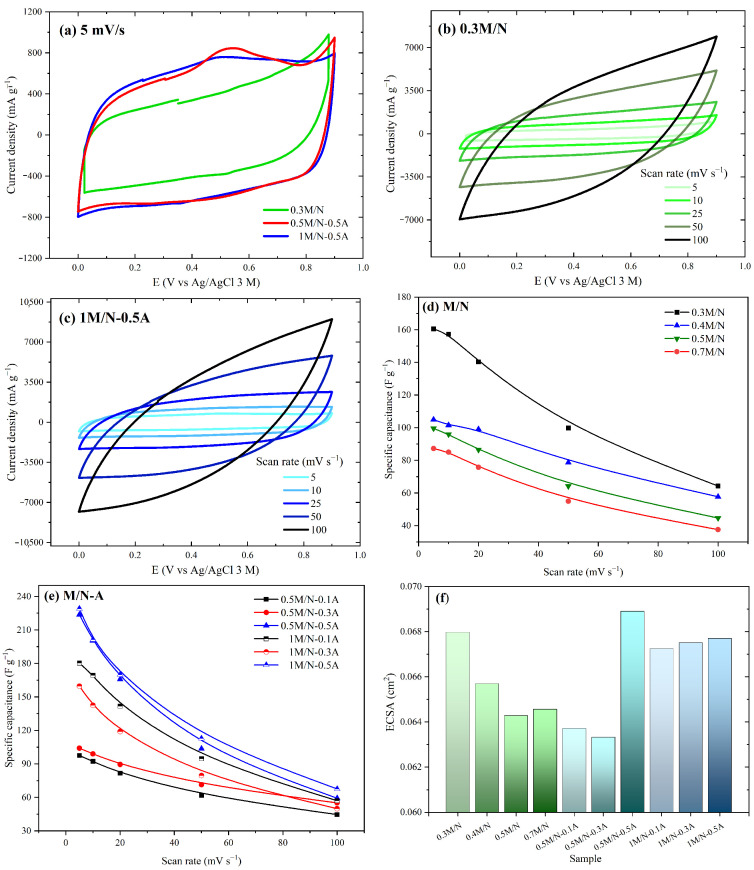
Electrochemical behavior of Na/Ag-containing ${\mathrm{Mn}}_{x}O_{y}$ composites in 0.1 M ${\mathrm{Na}}_{2}{\mathrm{SO}}_{4}$ by CV: (**a**) 5 mV $s^{-1}$; scan rates of (**b**) 0.3 M/N and (**c**) 1 M/N-0.5A. Specific capacitance plots at different scan rates of ${\mathrm{Mn}}_{x}O_{y}$ systems containing (**d**) Na and (**e**) Ag. (**f**) Electrochemically active surface area (ECSA) of the different electrodes.

The ion dynamics at the electrode–electrolyte interface of the Na/Ag-containing ${\mathrm{Mn}}_{x}O_{y}$ composites were studied by the CV technique. Figure 4 shows the CV curves of the electrode materials in 0.1 M ${\mathrm{Na}}_{2}{\mathrm{SO}}_{4}$ at varying scan rates ranging from 0 to 0.9 V vs. Ag/AgCl/NaCl [3 M]. The presence of peaks at Ep_1_ ≈ 0.56 V and Ep_2_ ≈ 0.26 V is evident, which is attributed to redox reaction potentials of Mn ions (see Figure 4a). These characteristic peaks are less noticeable in the 0.3 M/N and 1 M/N-0.5A systems, suggesting a fast insertion/de-insertion of sodium ions [15]. Additionally, the quasi-rectangular shape of the CV profiles indicates that the analyzed systems can store energy as pseudocapacitors; this characteristic is more evident in 0.5 M/N-0.5A and 1 M/N-0.5A than in 0.3 M/N. These results reveal that doping with a high concentration of ${\mathrm{AgNO}}_{3}$ improves the pseudocapacitive properties of ${\mathrm{Mn}}_{x}O_{y}$ systems. Figure 4b,c show CV profiles of 0.3 M/N and 1 M/N-0.5A in 0.1 M ${\mathrm{Na}}_{2}{\mathrm{SO}}_{4}$ at different scan rates, respectively. Increasing the scan rate resulted in area increase in the CV curve and modification of the quasi-rectangular shape of the CV profiles. Specific capacitance values ($C_{s,\mathrm{CV}}$) were determined from CV curves as follows:

(8) $$C_{s,\mathrm{CV}}=\frac{\int_{V_{2}}^{V_{1}}i\cdot dV}{v\cdot \Delta V\cdot m}$$

where *i* is the current, ΔV is the applied potential window ($\Delta V=V_{2}-V_{1}$), *v* is the voltage scan rate, and *m* is the mass of the electrode active material. The integral of Equation (8) was determined by using the internal area of each CV curve.

Figure 4d,e show the $C_{s,\mathrm{CV}}$ values as functions of the scan rate of Na and Na/Ag-containing ${\mathrm{Mn}}_{x}O_{y}$ composites in 0.1 M ${\mathrm{Na}}_{2}{\mathrm{SO}}_{4}$, respectively. As expected, increasing the scan rate reduced the $C_{s,\mathrm{CV}}$ values due to more efficient ion diffusion between the electrolyte and each site of the active material at low scan rate [15]. Figure 4d presents $C_{s,\mathrm{CV}}$ values of Na-containing ${\mathrm{Mn}}_{x}O_{y}$ composites, M/N systems, where the system with the lowest M/N ratio obtained the highest value of 160.5 F $g^{-1}$. In contrast, among M/N-A systems, the 0.5 M/N-0.5A and 1 M/N-0.5A systems at 5 mV $s^{-1}$ achieved higher $C_{s,\mathrm{CV}}$ values of 223.7 and 229.1 F $g^{-1}$, respectively (see Figure 4e). Sodium and silver ions contributed to the charge storage process by providing higher current density in these composites. These results are in good agreement with those reported by Gu et al., who indicated that ${\mathrm{Mn}}_{2}O_{3}$-based electrodes had higher specific capacitance than ${\mathrm{Mn}}_{3}O_{4}$-based electrodes [13].

In order to relate the electrochemically active surface area (ECSA) to the electrochemical behavior of the electrodes, the double-layer capacitance ($C_{dl}$) was first determined using CV experiments. $C_{dl}$ and ECSA values were estimated using Equations (9) and (10), respectively [48].

(9) $$C_{dl}=\frac{i_{a}-i_{c}}{2v}$$

(10) $$ECSA=\frac{C_{dl}}{C_{s}}$$

where $i_{a}$ and $i_{c}$ are anodic and cathodic current at ΔE = 0.44 V (non-faradaic potential window) and $C_{s}$ is in F ${\mathrm{cm}}^{-2}$. Figure 4f plots the results obtained from the different systems, revealing a clear trend. Decreasing the MnSO_4_·$H_{2}{\mathrm{O/NaNO}}_{3}$ ratio (M/N group) and increasing the ${\mathrm{AgNO}}_{3}$ concentration (M/N-A group) increased the ECSA values. The 0.3 M/N, 0.5 M/N-0.5A, and 1 M/N-0.5A systems presented the highest values; therefore, increasing the number of active sites in these systems improves electrochemical kinetics and enables high charge storage.

The main energy storage mechanisms of pseudocapacitor materials involve electrochemical reactions in the electrode bulk and on the electrode surface [5]. In the case of ${\mathrm{Mn}}_{x}O_{y}$ composites, it has been reported that an electron–proton transfer systems involve the insertion/de-insertion of alkali metal cations through a fast and reversible charge storage process, although reduction and oxidation reactions have been reported for ${\mathrm{Mn}}_{x}O_{y}$ nanocomposites [9,15]. For the 0.5 M/N-0.5A and 1 M/N-0.5A systems, the presence of ${\mathrm{Ag}}_{1.15}{\mathrm{Mn}}_{8}O_{16}$ allows this electrochemical process to be represented as follows:

(11) $$\begin{array}{c} {\mathrm{Ag}}_{1.15}{\mathrm{Mn}}_{8}O_{16}+{\mathrm{Na}}^{+}+e^{-}↔{\mathrm{Ag}}_{1.15}{\mathrm{Mn}}_{8}O_{16}\mathrm{Na} \end{array}$$

Additionally, sodium cations from the electrolyte can be adsorbed on the ${\mathrm{Ag}}_{1.15}{\mathrm{Mn}}_{8}O_{16}$ surface, which contributes to the charge–discharge process:

(12) $$\begin{array}{c} {\left({\mathrm{Ag}}_{1.15}{\mathrm{Mn}}_{8}O_{16}\right)}_{\mathrm{surface}}+{\mathrm{Na}}^{+}+e^{-}↔({\mathrm{Ag}}_{1.15}{\mathrm{Mn}}_{8}O_{16}\mathrm{Na}{)}_{\mathrm{surface}} \end{array}$$

### 3.3. Galvanostatic Charge–Discharge

**Figure 5:**
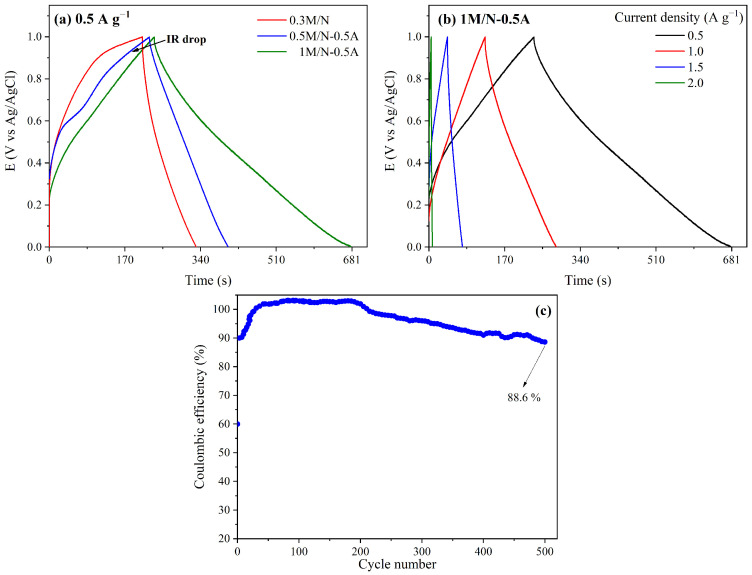
The GCD curves of (**a**) 0.3 M/N, 0.5 M/N-0.5A, and 1 M/N-0.5A systems at 0.5 A $g^{-1}$ and (**b**) 1 M/N-0.5A system at different current densities in 0.1 M ${\mathrm{Na}}_{2}{\mathrm{SO}}_{4}$. (**c**) The cyclic stability test of 1 M/N-0.5A at current density of 1 A $g^{-1}$.

Figure 5a illustrates the galvanostatic charge–discharge (GCD) profiles of the 0.3 M/N, 0.5 M/N-0.5A, and 1 M/N-0.5A systems within a potential range of 0.0 to 1.0 V and a current density of 0.5 A $g^{-1}$. The 1 M/N-0.5A stands out due to its relatively linear and semi-geometric behavior, which indicates high reversibility and high columbic efficiency. Additionally, a small IR drop is observed in the GCD profiles. This is associated with two resistances: $R_{ESR}$, which is related to the resistance of the electrolyte, cables, and electrode; and $R_{EDR}$, which is related to the resistance of ions accessing the outer pore of the electrode [49]. Therefore, at a current density of 0.5 A $g^{-1}$, the energy stored in the double electric layer during charging of the 0.5 M/N-0.5 A and 1 M/N-0.5 A systems is not lost during discharge. This reflects the good capacitive behavior and electrical conductivity of the Ag-containing ${\mathrm{Mn}}_{x}O_{y}$ composites. Furthermore, the 1 M/N-0.5A system exhibited a longer discharge time than the 0.3 M/N and 0.5 M/N-0.5A systems, suggesting that the presence of Ag and silver hollandite atoms enables higher specific capacitance.

Figure 5b shows the GCD profiles of the 1 M/N-0.5A system obtained at different current densities (from 0.5 to 2 A $g^{-1}$). As expected, the lower the applied current density, the longer the total discharge time ($t_{d}$). Using the $t_{d}$ values obtained and reported in Table 3, the specific capacitance by GCD ($C_{s,\mathrm{GCD}}$) was calculated according to the following equation [2]:

(13) $$C_{s,\mathrm{GCD}}=\frac{i\cdot t_{d}}{(\Delta V-IR)\cdot m}$$

where IR is the potential drop during discharge. The $C_{s,\mathrm{GCD}}$ values are consistent with those reported by the CV technique (see Table 3). The specific energy and power of the 1 M/N-0.5A system were also determined from the GCD tests using the relationships outlined by the Equations (14) and (15), respectively [2,50]:

(14) $$E=\frac{C_{s,\mathrm{GCD}}\cdot \Delta V^{2}}{2}$$

(15) $$P=\frac{E}{t_{d}}$$

The specific energy and power of the 1 M/N-0.5A system at a current of 0.5 A $g^{-1}$ were 31.3 Wh ${\mathrm{kg}}^{-1}$ and 253.8 W ${\mathrm{kg}}^{-1}$, respectively (see Table 1). These values are related to the good specific capacitance and rapid charge–discharge capacity in 0.1 M ${\mathrm{NaSO}}_{4}$ of the composite [50]. Finally, the Figure 5 shows the results obtained for the cyclic stability of the 1 M/N-0.5A system at 1 A $g^{-1}$. The system exhibits adequate cyclic stability during the initial cycles; however, an increase in the number of cycles results in a slight decrease in coulombic efficiency, reaching 88.6 % after 500 cycles.

### 3.4. Electrochemical Impedance Spectroscopy

**Figure 6:**
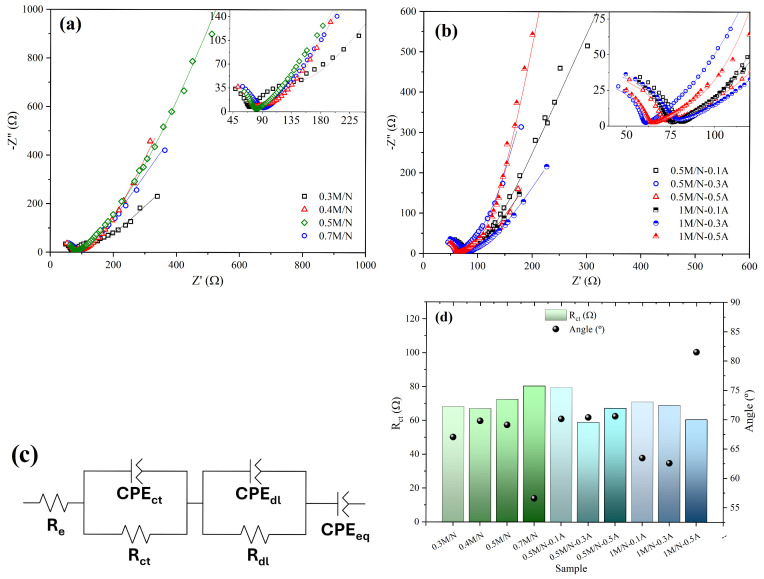
Electrochemical behavior of Na/Ag-containing ${\mathrm{Mn}}_{x}O_{y}$ composites in 0.1 M ${\mathrm{Na}}_{2}{\mathrm{SO}}_{4}$ by EIS: Nyquist plots of ${\mathrm{Mn}}_{x}O_{y}$ containing (**a**) Na and (**b**) Ag; (**c**) EEC used to fit the experimental data; (**d**) $R_{ct}$ and inclination angle values at low frequencies.

Figure 6 shows Nyquist plots of Na/Ag-containing ${\mathrm{Mn}}_{x}O_{y}$ composites in 0.1 M ${\mathrm{Na}}_{2}{\mathrm{SO}}_{4}$. High- (≈100 kHz) and low-frequency (≈0 mHz) regions are observed, which are related to low and high real impedance values, respectively. Similar impedance behavior was obtained for all the systems: at high frequencies, the impedance spectra present incomplete semicircles; at medium frequencies, linear behavior begins at an angle of approximately 45° with respect to the real impedance axis (x-axis); finally, at low frequencies, a linear tendency is maintained, and angle values are greater than 45°. In the high frequency region, the incomplete semicircles represent the retrieved internal resistance, which is the sum of the electrode and bulk electrolyte resistances [51,52], where the impedance value at the intersection of the semicircle and real impedance is related to the system’s ability to transport charge [51]. For M/N systems, the lowest MnSO_4_·$H_{2}{\mathrm{O/NaNO}}_{3}$ ratio (0.3 M/N) shows the lowest value, see Figure 6a; in contrast, the 1 M/N-0.5A and 0.5 M/N-0.5A systems (Figure 6b) achieved lower values at high silver concentrations, indicating a fast charge transport process, a desired characteristic in pseudocapacitors. In addition, at intermediate frequencies, the transition of the curves to linear behavior with 45° slope angles indicates that all reactive sites at the solid–liquid interface interact rapidly with the ions in the electrolyte [53]. The low-frequency region of the Nyquist plots shows lines with slope angles that are mostly greater than 45°. Ideal capacitors are known to exhibit linear behavior with an angle of 90°. Therefore, it can be concluded that the 1 M/N-0.5A system has superior pseudocapacitive properties compared to the rest of the systems [54].

In order to interpret quantitatively the EIS results and analyze the properties of these supercapacitive materials, the experimental data from the impedance spectra were fitted to an EEC, which combined capacitive and resistive elements (Figure 6c). The EEC is composed of the following electrical elements: an electrode resistance ($R_{e}$); an $RC_{ct}$ component corresponding to the charge transfer process at the current collector/active material interface, related to a resistor (*R*), and a constant phase element (*C*) that describes a pseudocapacitive element (*C*); an $RC_{dl}$ component related to the diffuse layer of the interface; and finally, equilibrium differential capacitance ($C_{eq}$) [51,52,55]. The electrical elements obtained from the different systems by the EEC are reported in Table 4. The low $R_{el}$ values suggest that the methodology used to prepare the working electrode was effective. The $C_{dl}$ values were calculated using the $CPE_{dl}$ and $R_{dl}$ components [56,57]:

(16) $$C_{dl}={\left(Y_{dl}R_{dl}^{1-n}\right)}^{\frac{1}{n}}$$

where $Y_{dl}$ and n are the proportionality factor and the exponent of the CPE, respectively. The 0.3 M/N, 0.5 M/N-A, 1 M/N-0.3A, and 1 M/N-0.5A systems presented the highest $C_{dl}$ values (see Table 4). As $C_{dl}$ contributes to the charge storage capacity of the electrode, it can be concluded that the low MnSO_4_·$H_{2}{\mathrm{O/NaNO}}_{3}$ ratio and high ${\mathrm{AgNO}}_{3}$ concentration improved the pseudocapacitive properties of these systems. The values of the proportionality factor of the equilibrium differential capacitance ($Y_{eq}$) related to the impedance response at low frequencies suggest lower capacitance dispersion in the analyzed systems. The $R_{ct}$ values obtained by the EEC are shown in Figure 6d. An increase in MnSO_4_·$H_{2}$O increased the $R_{ct}$ values for the M/N composite group. However, for the M/N-A systems, a larger amount of ${\mathrm{AgNO}}_{3}$ provoked a reduction in $R_{ct}$. For the last composite group, the charge transport process from the liquid phase (electrolyte) to solid phase (electrode material) was accelerated due to the increase in conductivity caused by Ag doping [4,52]. The lowest $R_{ct}$ value of 60.5 Ω was obtained with the 1 M/N-0.5A system, which elucidated its high specific power. Additionally, Figure 6d displays the inclination angles relative to the real impedance axis at low frequencies. The results suggested that the 1 M/N-0.5A system presented an angle greater than 81.$5^{\circ }$, confirming its lower capacitance dispersion [49]. Using a smaller amount of ${\mathrm{NaNO}}_{3}$ precursor during the molten salt synthesis process could favor the ${\mathrm{Mn}}_{2}O_{3}$ production with larger surface area, which facilitates the electron transfer [58].

## 4. Conclusions

The molten salt synthesis of Na/Ag-containing ${\mathrm{Mn}}_{x}O_{y}$ composites produced two groups of electrode materials: the first composite group synthesized without ${\mathrm{AgNO}}_{3}$ (M/N) mainly presented ${\mathrm{Mn}}_{2}O_{3}$, ${\mathrm{Mn}}_{3}O_{4}$, and ${\mathrm{Na}}_{0.31}^{1+}$(${\mathrm{Mn}}_{0.69}^{4+}{\mathrm{Mn}}_{0.31}^{3+}$)O_2_·$0.4H_{2}$O, while the second group, which was synthesized with ${\mathrm{AgNO}}_{3}$ (M/N-A), showed ${\mathrm{Mn}}_{2}O_{3}$, ${\mathrm{Ag}}_{1.15}{\mathrm{Mn}}_{8}O_{16}$, and Ag. SEM-EDS analysis confirmed uniform surface structures typical of Na/Ag-doped ${\mathrm{Mn}}_{x}O_{y}$ composites. The 0.5 M/N-0.5A and 1 M/N-0.5A systems exhibited the highest specific capacitances, 223.7 and 229.1 F $g^{-1}$, respectively. The incorporation of Ag improved electrochemical performance by enhancing charge transfer kinetics, conductivity, and electroactive surface area. In GCD profiles, the 1 M/N-0.5A system showed a near-linear and semi-symmetric shape, indicating high reversibility and coulombic efficiency. At 0.5 A $g^{-1}$, it delivered a specific energy of 31.3 Wh ${\mathrm{kg}}^{-1}$ and power of 253.8 W ${\mathrm{kg}}^{-1}$, attributed to its good capacitance and fast charge–discharge capability. Its cyclic performance at 1 A $g^{-1}$ remained stable in early cycles, with coulombic efficiency reaching 88.7% after 500 cycles. The presence of ${\mathrm{Ag}}_{1.15}{\mathrm{Mn}}_{8}O_{16}$ and Ag in the 1 M/N-0.5A system caused lower $R_{ct}$ values, as well as an inclination angle greater than 81.5° in the EIS spectra. This good electrochemical behavior was related to the low and high amounts of ${\mathrm{NaNO}}_{3}$ and ${\mathrm{AgNO}}_{3}$, respectively.

## Figures and Tables

**Table 1:** Synthesis conditions and abbreviations of Na/Ag-containing ${\mathrm{Mn}}_{x}O_{y}$ composites.

Sample Abbreviation	MnSO_4_·H_2_O/NaNO_3_	MnSO_4_·H_2_O (g)	${\mathrm{NaNO}}_{3}$ (g)	${\mathrm{AgNO}}_{3}$ (g)	NaOH (g)
0.3 M/N	0.3	3	9	0	3
0.4 M/N	0.4	4	9	0	3
0.5 M/N	0.5	5	9	0	3
0.7 M/N	0.7	6	9	0	3
0.5 M/N-0.1A	0.5	3	6	0.1	1
0.5 M/N-0.3A	0.5	3	6	0.3	1
0.5 M/N-0.5A	0.5	3	6	0.5	1
1 M/N-0.1A	1	3	3	0.1	1
1 M/N-0.3A	1	3	3	0.3	1
1 M/N-0.5A	1	3	3	0.5	1

**Table 2:** Elemental composition of Na/Ag-containing ${\mathrm{Mn}}_{x}O_{y}$ composites by EDS.

Sample	O	Na	Mn	Ag
0.3 M/N	69.53	5.69	24.78	0.00
0.5 M/N-0.5Ag	73.01	4.96	18.51	3.53
1 M/N-0.5Ag	72.22	3.4	19.34	5.04

**Table 3:** Results obtained from the 1 M/N-0.5A system in 0.1 M ${\mathrm{NaSO}}_{4}$ at 0.5 A $g^{-1}$ by GCD.

*I* (A $g^{-1}$)	$t_{s}$ (s)	$C_{s,\mathrm{GCD}}$ (F $g^{-1}$)	*E* (Wh ${\mathrm{kg}}^{-1}$)	*P* (W ${\mathrm{kg}}^{-1}$)
0.5	443.3	225.1	31.3	253.8
1.0	159.8	162.2	22.5	507.5
1.5	33.7	51.3	7.1	761.3
2.0	2.9	5.9	0.8	1015.0

**Table 4:** Impedance parameters of Na/Ag-containing ${\mathrm{Mn}}_{x}O_{y}$ composites in 0.1 M ${\mathrm{NaSO}}_{4}$.

Sample	$R_{\mathrm{el}}$ (Ω)	$C_{\mathrm{dl}}$ (mF)	$Y_{\mathrm{eq}}$ (mS $s^{n}$)	$n_{\mathrm{eq}}$	$R_{\mathrm{ct}}$ (Ω)	Angle (°)
0.3 M/N	0.083	46.8	14.4	0.84	68.2	67.0
0.4 M/N	0.017	20.3	3.3	0.72	67.1	69.7
0.5 M/N	0.869	12.1	7.3	0.86	72.5	69.1
0.7 M/N	0.021	18.0	4.1	0.62	80.4	56.5
0.5 M/N-0.1A	0.027	35.8	5.4	0.86	79.5	70.1
0.5 M/N-0.3A	0.003	28.9	6.1	0.91	59.0	70.3
0.5 M/N-0.5A	0.002	49.1	9.9	0.83	67.4	70.6
1 M/N-0.1A	0.000	18.8	8.0	0.81	71.1	63.4
1 M/N-0.3A	0.050	57.5	7.9	0.77	69.1	62.6
1 M/N-0.5A	0.083	41.7	22.0	0.95	60.6	81.5

## Data Availability

The data presented in this study are available on request from the corresponding author.
